# The Effects of GABAergic System under Cerebral Ischemia: Spotlight on Cognitive Function

**DOI:** 10.1155/2020/8856722

**Published:** 2020-09-28

**Authors:** Juan Li, Luting Chen, Feng Guo, Xiaohua Han

**Affiliations:** ^1^Department of Rehabilitation Medicine, Tongji Hospital, Tongji Medical College, Huazhong University of Science and Technology, Wuhan, Hubei, China; ^2^Department of Rehabilitation Medicine, General Hospital of the Yangtze River Shipping, Wuhan, Hubei, China

## Abstract

In this review, we present evidence about the changes of the GABAergic system on the hippocampus under the ischemic environment, which may be an underlying mechanism to the ischemia-induced cognitive deficit. GABAergic system, in contrast to the glutamatergic system, is considered to play an inhibitory effect on the central nervous system over the past several decades. It has received widespread attention in the area of schizophrenia and epilepsy. The GABAergic system has a significant effect in promoting neural development and formation of local neural circuits of the brain, which is the structural basis of cognitive function. There have been a number of reviews describing changes in the GABAergic system in cerebral ischemia in recent years. However, no study has investigated the changes in the system in the hippocampus during cerebral ischemic injury, which results in cognitive impairment, particularly at the chronic ischemic stage and the late phase of ischemia. We present a review of the changes of the GABAergic system in the hippocampus under ischemia, including GABA interneurons, extracellular GABA neurotransmitter, and GABA receptors. Several studies are also listed correlating amelioration of cognitive impairment by regulating the GABAergic system in the hippocampus damaged under ischemia. Furthermore, exogenous cell transplantation, which improves cognition by modulating the GABAergic system, will also be described in this review to bring new insight and strategy on solving cognitive deficits caused by cerebral ischemia.

## 1. Introduction

Cerebral ischemia is hypoperfusion of the blood through brain tissue caused by a pathologic constriction or obstruction of its blood vessels or an absence of blood circulation. Both transient and chronic cerebral ischemia or chronic cerebral hypoperfusion can lead to damage or death of brain cells and other pathophysiological changes. Meanwhile, cerebral hypoperfusion is considered a major cause of vascular dementia (VD) [1] and an underlying pathological mechanism of Alzheimer's disease (AD) [2]. Thus, one of the main threats of cerebral ischemia is the impairment of cognitive function. The GABAergic system of the mammalian brain consists of GABA-releasing cells and receptors that bind GABA [3]. A growing body of research in recent decades has shown that the GABAergic system is strongly associated with cognitive function, which plays an important role in neurological diseases, such as VD [4], AD [5], depression [6], and schizophrenia [7]. However, ischemia can cause damage to the GABAergic system in brain regions that are related to cognitive function, such as the hippocampus, amygdala, and prefrontal cortex [8–10]. Therefore, the impairment of the GABAergic system is considered one of the mechanisms affecting cognition. Over the past few years, with extensive studies about the relationship between the hippocampus and cognitive function, the hippocampus has been observed to play a central role in the cognitive map. This means that the brain builds a unified representation of the spatial environment to support memory and guide future action [11]. Thus, this review focuses on the effect of cerebral ischemia on cognitive disorder induced by the deficit of the GABAergic system. We describe the changes of the GABAergic system on the hippocampus under the condition of ischemia and summarize some of the evidence that can improve cognitive function by regulating the GABAergic system of the hippocampus. Furthermore, as cell transplantation has played a significant role in almost every medical field in recent years, we introduce some studies about cell transplantation to improve cognition via the contribution of the GABAergic system.

## 2. The Overview of the GABAergic System

GABAergic system involves GABA-releasing cells, the biosynthesis and metabolic degradation of GABA, its release, and interaction with receptors. In the hippocampus, GABAergic interneurons are in the minority and account for about 10–15% of the total number of neurons. They make a vast difference in all aspects of cortical circuit function and regulation because of its remarkable diversity, extensive distribution, and intimate contact with pyramidal cells [12]. On the basis of the expression of specific molecular markers, according to the molecular classification, the GABAergic interneurons can be divided into five main groups: parvalbumin (PV), somatostatin (SOM/SS), neuropeptide Y (NPY), vasoactive intestinal peptide (VIP), and cholecystokinin (CCK) interneuron [13]. Gamma-aminobutyric acid (GABA), as a chief inhibitory neurotransmitter in the developmentally mature mammalian central nervous system, is synthesized from glutamic acid by glutamic acid decarboxylase (GAD), which has two GAD subtypes, GAD65 and GAD67. GABA is stored in synaptic vesicles, released in synaptic cleft triggered by the nerve impulse, and then interacts with its receptors. There are three different classes of GABA receptors, namely, ionotropic GABAA and GABAC receptors and metabotropic GABAB receptors in the central nervous system [14, 15]. Of these, GABAA and GABAB receptors have received the greatest amount of attention in cerebral ischemia [16, 17]. The GABAA receptors are ligand-gated pentameric chloride channels and composed from a variety of subunits that include *α* (subtypes 1–6), *β* (1–4), *γ* (1–3), *δ*, *ε*, *π*, and *ρ* which have different sensitivities to GABA and modulatory drugs [18]. Furthermore, the GABAA receptors have two main modes of operation, phasic and tonic. The phasic effects of GABA are seen in GABAergic synapses, whereas the tonic inhibition is through extrasynaptic receptors that report GABA as a volume transmitter and set an excitability threshold for cortical neurons [3]. The GABAA receptors found extrasynaptically are comprised of relatively rare subunits, particularly *α*4, *α*6, and *δ*. Generally, *δ*-subunit containing the GABAA receptors are extrasynaptic, but vice-versa is not true [19]. *α*5*β*1*γ*2 is located postsynaptically, but *α*5*β*2*γ*2 and *α*5*β*3*γ*2 isoforms are extrasynaptic [20]. As a kind of metabolic receptor, GABAB receptor localizes in both the presynaptic and postsynaptic terminals, which has two subunits, GABAB1 and GABAB2 [21]. GABA transporters (GAT1–4) remove GABA from the synaptic cleft. This is followed by its catabolization by GABA transaminase (GABA-T), an enzyme that converts brain GABA into succinate semialdehyde, which can be converted to succinic acid and enters the citric acid cycle.

## 3. Effects of Cerebral Ischemia on the GABAergic System: Hippocampus

After cerebral ischemia, numerous studies have shown that a number of changes happened on the GABAergic system at the hippocampal area using in vivo models and in vitro oxygen-glucose deprivation (OGD) experiment. From the survival and function of GABA interneurons and intracellular and extracellular levels of the GABA neurotransmitter, to GABA signal transmissions, cerebral ischemia can induce the process of pathological variations on the GABAergic system as discussed below.

### 3.1. GABA Interneurons

Although some reports showed that the GABA interneurons were insensitive to ischemia, compared with principal neurons [22], several others have shown that the interneurons in the hippocampus are vulnerable to ischemia. For example, in addition to excitatory cells, SS- and NPY-expressing interneurons were lost from the dentate hilus in the days following ischemic injury. Simultaneously in CA1, the number of neurons containing only NPY decreased, while the number of neurons expressing both NPY and SS increased as the interneurons changed their patterns of peptide expression [23]. The PV immunoreactivity was unchanged up to two days after ischemia. However, at five and 14 days after ischemia, a conspicuous reduction of PV immunoreactivity was observed in interneurons of the hippocampal CA1 sector. Furthermore, a significant decrease in PV immunoreactivity was found in the interneurons of the hippocampal CA3 sector [24]. In another study, following transient cerebral ischemia (bilateral carotid occlusion for 2 min) in the gerbil, the GABAergic interneurons (labeled the GABAA receptor a1-subunit) developed severely beaded dendrites after 3–4 days throughout all layers of area CA1, and the varicose and fragmented appearance of a1-subunit-positive dendrites up to five weeks after ischemia was observed. However, there were no ischemia-induced changes in dendrites immunolabeled with the a1-subunit within area CA3 or the dentate gyrus [25]. On the contrary, a larger population of spiny interneurons disappeared from the hilus (feedback inhibitory interneurons) and stratum lucidum of CA3 (feedforward inhibitory interneurons) in a complete forebrain ischemia model induced by four-vessel occlusion after 12–14 months [26]. Since there are different ideas about whether the hippocampal GABA interneurons are resistant to ischemia, there are several possible explanations for this discrepancy. These include (1) a remarkable diversity of the GABA interneurons contributing to their functional versatility in shaping the spatiotemporal dynamics of neural circuit operations underlying cognition, which, in turn, demonstrate different sensitivities to ischemia within the hippocampus. Perhaps, the detection markers that represent the GABA interneurons are distinct. They are GABA, GAD67/65, GABA receptors, PV, SST, and so on. Some markers cannot replace all of the GABA interneurons [27]. Furthermore, the expression of these markers is regulated under the pathological process of cerebral ischemic injury. For example, one study showed neurons coexpressing SS and NPY before ischemia added to the number of neurons containing SS alone after ischemia [23]. However, the different reaction to ischemia brings a distinct outcome. During the hypoxic condition, the interneurons that expressed HIF-1*α* are more tolerant to a severe environment [28]. Another study showed that resistance of the interneurons to ischemic damage could be related to a lower expression level of pH-sensitive leakage potassium channels (TASK) currents compared to pyramidal cells [29]. (2) GABA interneurons have a different metabolic system from principal neurons. For instance, the GABA shunt is another energy metabolic pathway in addition to the Krebs cycle, which plays a critical role in cerebral hypoperfusion to produce ATP to help the interneurons survive (we will discuss this below). (3) GABA interneurons in different regions of the hippocampus have different sensitivity to ischemia. With changes in peptide expression patterns, the interneurons in the CA1 subfield seem more likely to survive compared to the dentate gyrus and CA3 subfield [26]. (4) Different durations of ischemia lead to a diverse hostile environment. As described above, there are different ischemic models, such as transient ischemia and chronic hypoperfusion, and a distinct duration of vascular occlusion. However, due to different experimental purposes, the detection time of the GABA interneurons after ischemia was different, which means that the interneurons may experience pathological states, from injury to loss. Even though GABA interneurons in the hippocampus have a certain tolerance to ischemia compared with the principal cells, however, long-term and chronic ischemia can affect its substructure and function. For example, Zhan et al. showed that the excitability of interneurons in CA1 declined due to impaired Na+ channel activation in the transient cerebral ischemia model, and this may be one of the reasons for excitotoxicity that contributes to pyramidal cell death [30].

### 3.2. GABA Neurotransmitter

GABA, as the opposite of excitatory neurotransmitter-glutamate, is crucial for normal neurologic function [31]. At the initial stage of ischemia, the toxicity of enhanced excitatory amino acid function was thought to be one of the causes of cell death, and enhancing extracellular GABA levels had neuroprotective effects at an early time [32]. However, extracellular GABA levels varied significantly in different periods of cerebral ischemia. One study showed an increase of GABA after the ligation of the bilateral carotid artery for 5 min and reperfusion for 60 min in the gerbil hippocampus [33]. Huang et al. showed that in the permanent middle cerebral artery occlusion (pMCAO) model and the transient cerebral focal ischemia (tMCAO) model, the levels of GABA decrease after seven days [34]. Another research showed that GABA in the hippocampal CA1 subfield was decreased significantly after one month in permanent bilateral occlusion of the common carotid arteries (two-vessel occlusion, 2VO) procedures [35]. GAD, GAT, and GABA-T, as synthesis, transport, and decomposition tools of GABA, respectively, can affect the level of GABA when they have a change during cerebral ischemic injury. However, just like the temporal alteration of GABA, some of them got significantly altered in the CA1 region after ischemia. The immunoreactivities of the GAD isoforms were markedly elevated in the CA1 region at 30 min postischemia, then recovered to baseline at 3 h, but the intensity of GAD67, and not GAD65, markedly increased at 24 h postischemia. GAT-1 expressions were elevated in the CA1 region at 12 h postischemia, which were involved in the reverse transport of GABA, not reuptake, to enhance the level of extracellular GABA, as an inhibitory neurotransmitter. Meanwhile, the GABA-T immunoreactivity in the CA1 area decreased simultaneously. In contrast, at 24 h postischemia, both GAT-1 (involved in reuptake of GABA) and GABA-T expressions in the CA1 area was enhanced, which was considered to be a degradation of GABA as a neurotransmitter, but enhancement as a metabolite [36]. Thus, these changes may explain why extracellular GABA levels increase during early ischemia and decrease at the chronic phase (we will discuss below). Importantly, all of those abnormal changes would have a great influence not only on the hippocampus and its local circle but also on other brain regions. For example, GAD67 deficiency in parvalbumin interneurons produces deficits in inhibitory transmission and network disinhibition in the mouse prefrontal cortex [37]. For the changes of extracellular GABA levels at different times of ischemia, Rochelle et al. summarized several mechanisms for an early increase in extracellular GABA during ischemia [38]. About the late decrease, several explanations can be proposed, includingthe following: (1) the dysfunction of GABA interneurons, which release GABA as their neurotransmitter. The GABA interneurons show their insensitivity to the early hypoperfusion (just like we discuss above), but their substructure and function would be damaged with the extension of ischemic time. (2) The decline of release and reuptake of GABA [39]. Volgyi et al. showed that GABAergic synaptic transmission-related proteins, sodium- and chloride dependent GABA transporter 1 (SLC6A1), and GABA type B receptor subunit 2 (GABABR2), decreased during chronic cerebral hypoperfusion [40]. (3) The GABA shunt: in addition to being a neurotransmitter, GABA is also a metabolic substance. The GABA shunt is a conserved energy metabolic pathway, which generates succinate from amino acids and thus is an anaplerotic pathway to the Krebs cycle to produce ATP [41]. The GABA shunt consists of three enzymatic reactions catalyzed by glutamate decarboxylase (GAD), GABA transaminase (GABA-T), and succinic semialdehyde dehydrogenase (SSADH), which can be activated in cerebral hypoperfusion [34, 36, 40, 42]. During the chronic phase, to adapt the state of glucose deficiency and hypoxia, the GABA interneurons change their energy metabolic pathway to acquire ATP. So, this may be one of the reasons the GABA interneurons survive, but also the reason for the lower level of GABA at a later phase. In general, the change of GABA levels is to accommodate the pathological process of ischemia in the brain.

### 3.3. GABA Receptors

It is well known that both GABAA and GABAB receptors play a big role in cognition. After hypoperfusion, in addition to the normal level of GABA neurotransmitters, GABA signal transmissions also play an important role. However, a number of studies have shown that the expression of both GABAA and GABAB receptors was generally decreased in the acute and chronic phases [10, 25]. For example, in one study, animals treated with 2VO procedures showed that the expression of GABAB receptor 1 (GABABR1) in the hippocampal CA1 subfield was decreased significantly after one month [35]. Similarly, in another study, there was a marked decrease in both mRNA and protein expression of GABA subtypes (GABAA and GABAB) in different brain regions of rats at 30 days after 2VO, especially in the hippocampus [43]. In an OGD study to investigate the alteration of the protein levels of the GABAB1 and GABAB2 receptor subunits in rat organotypic hippocampal slice cultures by ischemia-like challenges, the result showed a marked decrease in the total levels of GABAB2 (~75%), while there was no significant change in the levels of GABAB1 after 24 h [44]. Furthermore, there was a difference in the expression of subunits and the location of receptors. In in vitro experiment, exposure of hippocampal slices to OGD for 90 min shows downregulation of all the synaptic GABAAR subunits of ~40% for *α*1 subunits, ~20% for *α*2 subunits, and~35% for *β*3 and *γ*2 subunits, but no effect was found for the *δ* subunit [45]. Liu et al. show that five weeks after induction of hypoperfusion, the surface expression of GABAA receptor *α*1 subunit was significantly decreased, but an intracellular expression of GABAA receptor *α*1 subunit was significantly increased [46]. Thus, the change of GABA receptors after ischemia is not only an expression of decline but also involves selective expression of subunits and stability of localization. Mele et al. showed that the internalization of GABAAR was dependent on glutamate receptor activation and mediated by dephosphorylation of the *β*3 subunit at serine 408/409, and the expression of phosphomimetic mutant GABAAR *β*3 subunits prevented receptor internalization and protected hippocampal neurons from ischemic cell death [45]. Therefore, this is one of the reasons why GABA receptors decrease in the early stage of ischemia and phosphorylation of Ser408/409 in the GABAA*β*3 subunit, and Ser892 in the GABAB 2 subunit will increase the induction rate and magnitude of LTP at the hippocampus in 2VO rats [43].

In conclusion, the GABA interneurons may keep relatively intact cellular morphology after cerebral ischemia, but the substructural integrity of GABA interneurons, the normal level of extracellular GABA, and the natural function of GABA receptors would be required for regular functioning of a network such as the hippocampus.

## 4. Improvement of Cognitive Function by Regulating GABAergic System after Cerebral Ischemia

Just like we summarized above, cerebral ischemia has a huge effect on the GABAergic system, especially in the hippocampus, including loss or dysfunction of the GABA interneurons, abnormal levels of GABA neurotransmitter, selective patterns of expression, and decreased activity on GABA receptors. Numerous studies have shown that the GABAergic system has a strong relationship with cognitive function. Therefore, regulating the GABAergic system in the hippocampus after cerebral ischemia is another way to improve cognition. Meanwhile, neuroprotection during ischemia in the hippocampus, which can be modulated via the GABAergic system, contributes a lot to cognitive function [32]. For this reason, it will be discussed in this section.

### 4.1. Neuroprotection on the Hippocampus by Regulating GABAergic System

As already established, the hippocampus presents a critical role in cognitive function. Neurons of the hippocampus, as the basic structure or constitution, serve its local circuitry and process information. Since the loss of hippocampal neurons would induce the cognitive deficits [47], specifically, neuron loss in the hippocampus is one of the primary pathological processes of AD [48], which is the most common type of dementia. Thus, protecting the hippocampal neurons from damage caused by ischemia is an important strategy to reduce cognitive damage. Muscimol and baclofen, as GABA A receptor and GABA B receptor agonist, respectively, are often used in in vivo and in vitro experimental studies. In an in vivo study, muscimol and baclofen were coapplied on a brain ischemia model induced by the four-vessel occlusion (4-VO). The result showed that this intervention markedly decreased the neuronal loss in the hippocampal CA1 region. Interestingly, another discovery in this experiment was that the protection of baclofen was much weaker than muscimol [49]. This neuroprotective effect on the hippocampus by working on receptors can be found in a variety of ischemia models, including middle cerebral artery occlusion (MCAO) [50], transient brain hypoperfusion [51], chronic cerebral hypoperfusion [46], and OGD [52]. There are several mechanisms to explain the neuroprotective effects by regulating the GABAergic system: (1) the inhibition of apoptosis: a study showed that coactivation of the GABA A receptor and GABA B receptor triggered the additive neuroprotection to the hippocampal CA1 neurons by activation of the PI-3K/Akt pathway and inhibiting the ASK1-c-Jun N-terminal protein kinase cascade [49]. As direct Akt substrates, glycogen synthase kinase 3 (GSK-3) would be enhanced after cerebral hypoperfusion [53]. This process was involved in the pathological process of apoptosis and led to cognitive problems [54]. However, GSK-3 would be suppressed upon phosphorylation by Akt. (2) The recovery of ATP: diazepam, another agonist of GABA A receptor, was applied to hippocampal slices of adult rats exposed to OGD, showing that diazepam could completely restore ATP and prevented releasing of cytochrome c from the mitochondria [55]. Because this could promote caspase-3 activation, which led to apoptosis of neurons, this could be another way to inhibit apoptosis. (3) The inhibition of autophagy: baclofen was applied in a chronic cerebral hypoperfusion model, and the results showed that activation of GABAB receptors suppressed not only cytodestructive autophagic activity through Akt/ERK-Bcl2-Beclin1 signaling pathway but also upregulated protective autophagy through the activation of the GABAA receptor-CX43/CX36 signaling pathway [46]. (4) Resistance to excitatory toxicity: coactivation of the GABA A receptor and GABA B receptor by muscimol and baclofen in rat 4-VO ischemic model showed that the intervention protected neurons from neuronal death through downregulating the function of NMDA receptors via attenuating the tyrosine phosphorylation of NR2A subunit [56]. In another OGD study, activating the GABAA receptor by JM-1232(-) (JM) reduced the elevation of intracellular Ca2+ concentration during OGD [57]. In summary, neuroprotection on the hippocampus is extremely crucial because the structure of the hippocampus is the foundation of its function. Thus, applying GABAergic drugs to resist various death pathways should be the treatment of choice.

### 4.2. Improvement of Cognitive Function by Regulating GABAergic System

As we discussed above, cerebral ischemia leads to loss or dysfunction of the GABA interneurons, abnormal levels of GABA neurotransmitter, selective patterns of expression, and decreased activity on GABA receptors. Considering the difficulty of restoring the lost interneurons and promote the release of GABA neurotransmitter, modulating the activity of GABA receptors is more feasible. Because GABAA receptors have two main modes of operation, phasic and tonic, these two receptors mediate different physiological processes in normal circumstances. However, in the pathological process of ischemia, the two receptor-mediated effects are quite different. For example, although activating the GABAA receptor has a neuroprotective effect, however, the application of S44819, a kind of selective extrasynaptic *α*5-GABAA receptor inhibitor, can improve cognitive performance in preclinical models of vascular cognitive impairment (VCI) induced by permanent occlusion of the right common carotid artery (rUCO) [58]. However, dampening tonic inhibition too early after stroke may produce an opposite effect, that is, increased cell death [58, 59]. Collectively, drugs that selectively target the GABAA receptors subtype to interfere with GABAergic neurotransmission appear to be a promising strategy to facilitate poststroke recovery and/or to prevent cognitive deficits. The GABAB receptor is another potential site to play the role of cognitive enhancement. Baclofen, a GABAB receptor agonist, markedly improved the memory impairment and alleviated neuronal damage induced by 2VO after five weeks. The mechanism was that baclofen attenuated the decrease of surface expression of GABAB R1 and GABAB R2 and restored the balanced surface expression of HCN1/HCN2, which coregulated neuronal excitability with GABA receptors in the rat hippocampal CA1 area [4]. In another research, baclofen ameliorated cognitive deficits 2VO in rats by improving BDNF signaling and reverse Kir3 channel surface expressions in the hippocampal CA1 [60]. It has been proved that activation of GABA(B) receptors triggers the secretion of BDNF and promotes the maturation of GABAergic synapses in the newborn mouse hippocampus [61]. Furthermore, BDNF signaling plays an important role in the hippocampal long-term potentiation (LTP) and synaptic plasticity [62]. At 180 days posttransient cerebral ischemia, endogenous neural progenitor cells were found to differentiate into new GABAergic neurons, labeled glutamic decarboxylase 67 (GAD67), via the BDNF-TrkB pathway. Simultaneously, the new GABAergic neurons partially mediated the recovery of cognitive impairments [63]. Interestingly, clonidine, an *α*2-adrenergic receptor agonist, could ameliorate cognitive deficits and neuronal impairment induced by chronic cerebral hypoperfusion via the upregulation of GABABR1 and GAD67 in the hippocampal CA1. This may be related to the simultaneous release of GABA by stimulating adrenal receptors [64].

## 5. The Effect of Exogenous Neural Stem Cells on the GABAergic System

In recent years, cell transplantation technology is growing more promising. Interestingly, despite being difficult to operate, this technology brings new revelations. The neural stem cell transplantation can replace cells that have been lost or have lost their function, due to its inherent ability to differentiate into various cell phenotypes. Further, the transplanted cells can secrete combinations of trophic factors that modulate the molecular composition of the environment to evoke responses from resident cells [65, 66]. Because the GABAergic system suffers damage under cerebral ischemia, resulting in its dysfunction, regulating the GABAergic system and recovering its normal function become another method to protect neurons and improve cognition, as summarized above. However, those drugs work on receptors, which cannot repair damaged neurons. To compensate for the loss of the GABA interneurons, cell transplantation technology is conspicuous. In a study, phencyclidine (PCP), a noncompetitive NMDA receptor antagonist, was used to cause dysfunction of the GABAergic inhibitory interneurons in the prefrontal cortex (PFC) and cognitive deficits. Tanaka et al. showed that transplanting embryonic medial ganglionic eminence (MGE) cells, which would differentiate into a specific class of the GABAergic interneurons into prefrontal cortex (PFC), could prevent the induction of cognitive and sensory motor gating deficits by PCP. Specifically, the preventive effects were not reproduced by either transplantation of cortical projection neuron precursors into the mPFC or transplantation of MGE cells into the occipital cortex. So, the specific cell type damage in this area led to cognitive deficits and needed the specified cell type to repair in the right place [67]. Recent literature shows that causing the GABAergic interneuron impairments and aberrant neuronal activity in the hippocampal by apolipoprotein (apo) E4 and amyloid-*β* (A*β*) peptides might be another pathological process in AD-related mouse models and humans which could cause learning and memory deficits [68]. Tong et al. transplanted embryonic interneuron progenitors into the hippocampal hilus of aged apoE4 knockin mice with or without A*β* accumulation. The result was that transplantation of inhibitory interneurons developed into mature interneurons, functionally integrated into the hippocampal circuitry, and restored normal cognitive function in two widely used AD-related mouse models [69]. Furthermore, to ensure therapeutic benefits of transplanting exogenous neural stem cells and better adapt to the new environment, Martinez-Losa et al. made a genetic modification—Nav1.1-overexpressing on transplanted cells derived from the embryonic medial ganglionic eminence (MGE), many of which differentiated into the GABAergic interneurons in situ. The result showed that the Nav1.1-overexpressing group enhanced behavior-dependent gamma oscillatory activity, reduced network hypersynchrony, and improved cognitive functions in human amyloid precursor protein- (hAPP-) transgenic mice, which simulated key aspects of AD, compared to the wild group [70]. Importantly, in addition to the function of replacement, exogenous neural stem cells also showed their paracrine actions, which released a wide array of trophic factors that drove the endogenous cell repair. Transplantation of BMSCs was capable of improving cognitive impairment via upregulating the hippocampal GABAergic system in a rat model of chronic cerebral hypoperfusion (via upregulating hippocampal GABA, GAD67, and GABABR1 expression in a rat model of chronic cerebral hypoperfusion) [35, 71]. In a traumatic brain injury treatment (TBI) model, transplantation of neural stem cells (NSCs) could effectively alleviate the formation of the glial scar, improve the survival rate of the hippocampal neurons, and improve the cognitive dysfunction in rats after TBI. The underlying mechanism may be related to NSCs' effects on inhibiting the release of Glu and maintaining the content of GABA [72]. The transplantation of exogenous neural stem cells could repair the GABAergic system in the damaged area and alleviate cognitive dysfunction. Since the GABAergic system is impaired by cerebral ischemia, transplantation of neural stem cells will be another new and feasible therapeutic method to improve the cognitive function after cerebral ischemia.

## 6. Conclusion

In this review, we summarized the changes of the GABAergic system on the hippocampus under ischemia, mainly including the substructural damage and some permanent loss of the GABA interneurons, time varyingly extracellular GABA neurotransmitter, and dysfunctional GABA receptors. We also outlined the evidence that GABAergic function decreased following ischemia to cause cognitive impairment, but it can be ameliorated by regulating the GABAergic system in numerous animal experiments. In this section, underlying mechanisms involve the protection of neurons in the hippocampus and the regulation of abnormal GABA signaling pathways, such as activating synaptic GABAA receptors and inhibiting the extrasynaptic receptors. But only a few clinical studies have demonstrated that cognitive impairment caused by ischemia is alleviated by the use of GABAergic drugs. The side effects of GABAergic drugs might limit their use in improving cognition [73]. Also, the exogenous cell transplantation may improve cognition by modulating the GABAergic system in many animal models. However, the animal research involved in transplantation of neural stem cells to rescue the cognitive deficits caused by cerebral ischemia is insufficient. However, it brings us new insight and strategy to solve this tremendous obstacle of the cognitive deficits caused by cerebral ischemic injury.

## Data Availability

No data were used to support this study.
